# Mycobacterium abscessus - an uncommon, but important cause of peritoneal dialysis-associated peritonitis – case report and literature review

**DOI:** 10.1186/s12882-020-02146-4

**Published:** 2020-11-17

**Authors:** Anup Singh Jheeta, Jayakeerthi Rangaiah, John Clark, David Makanjuola, Subash Somalanka

**Affiliations:** 1 South West Thames Renal & Transplantation Unit, Epsom & St Helier University Hospitals NHS Trust, Wrythe Lane, Carshalton, Surrey, SM5 1AA UK; 2 Department of Microbiology, Epsom & St Helier University Hospitals NHS Trust, Wrythe Lane, Carshalton, Surrey, SM5 1AA UK

**Keywords:** Peritoneal dialysis, Catheter, Peritonitis, Mycobacterium abscessus

## Abstract

**Background:**

Peritoneal dialysis (PD) is a form of therapy for end-stage kidney disease (ESKD), and peritonitis is a known complication. *Mycobacterium (M)* species associated peritonitis in PD patients is uncommon. Our experience of managing PD associated peritonitis caused by *M abscessus* in a middle-aged man with ESKD due to focal segmental glomerulosclerosis is shared in this article with a review of the literature on this condition.

**Case presentation:**

A 49-year old man presented to our unit with symptoms of peritonitis and cloudy PD effluent. Initial analysis of PD fluid showed Gram stain was negative, with no organism grown. Empirical PD peritonitis treatment with intra-peritoneal antibiotics did not improve his symptoms and he required intravenous antibiotics, PD catheter removal and a switch to haemodialysis. Cultures of the PD fluid later grew *M abscessus,* and the antibiotic regimen was changed appropriately, leading to clinical improvement.

**Conclusion:**

*M abscessus* associated peritonitis in PD patients is rare. It needs to be borne in mind when clinical improvement is not seen with standard broad-spectrum antibiotics, especially in situations where the PD fluid is initially deemed to be culture negative. PD fluid samples should be sent for acid-fast bacillus and if detected, should be further analysed with genome-wide sequencing to confirm the species of the *Mycobacterium.* Prompt removal of the catheter with peritoneal washout is critical for clinical improvement.

## Background

Peritoneal dialysis is used for managing end-stage kidney disease (ESKD). It requires access to the peritoneal space by way of a percutaneous tunnelled dialysis catheter. Infective complications of peritoneal dialysis (PD) include catheter exit-site infection (ESI), catheter tunnel infection (TI) and peritonitis. Peritonitis can be particularly severe and can lead to PD failure and discontinuation of PD. [1] Early recognition of infections, prompt microbiological diagnosis, and establishing appropriate treatment are therefore essential. An essential factor that determines a successful outcome in such cases is anti-microbial susceptibility testing to guide appropriate therapy. PD related peritonitis is most commonly caused by gram-positive aerobes such as coagulase-negative *Staphylococci* and *Staphylococcus aureus,* or gram-negative aerobes such as *Pseudomonas aeruginosa* [2]. Reports of PD related peritonitis caused by *Mycobacterium* species are relatively rare, although their incidence has been well documented [3, 4]. Nontuberculous mycobacteria (NTM) are members of the genus *Mycobacterium* but are significantly different from other members of the same genus, e.g., *M.tuberculosis* and *M.leprae.* NTM related PD peritonitis is emerging as a diagnostic and therapeutic challenge in the management of PD peritonitis, with comparatively higher rates of PD catheter loss and PD failure compared to other organisms [5]. Consensus on the management of NTM related PD peritonitis does not currently exist, possibly because of the paucity of reported cases. We describe what is to our knowledge the first reported case in the UK of PD related peritonitis secondary to the NTM species *Mycobacterium abscessus.*

## Case presentation

A 49-year-old male of Afro-Caribbean origin with a background of hypertension, type 2 diabetes mellitus, high body mass index of 40 and ESKD secondary to focal and segmental glomerulosclerosis (FSGS) diagnosed in 2007 and received only 1 month of corticosteroids. He presented to our PD unit with mild abdominal discomfort and cloudy PD effluent in July 2019. He had been on PD for 4 years and had experienced one previous episode of PD peritonitis caused by *Staphylococcus aureus* requiring catheter replacement 2 years ago. The PD regimen consisted of automated peritoneal dialysis [APD] of 8.5 h duration with 5 cycles of 3 l fills. The dialysate fluid consisted of 1.36% glucose concentration of 10 l and 2.27% glucose concentration of 5 l with extraneal (Icodextrin 7.5%) solution as a last fill of 1.7 l. He also received an Opti exchange of 2.0 l of 1.36% solution in the evenings.

He was clinically and haemodynamically stable with mild abdominal discomfort as his symptom. Serum inflammatory markers showed a white cell count [WCC] of 6.0 × 10^6^/L and a C-reactive protein [CRP] of 80.7 mg/L (normal range < 5 mg/L). Microscopy of the PD fluid showed a WCC of 155 x 10^6^cells/L and a total eosinophil count of 1 × 10^6^ cells/L, but the Gram stain was negative. Empirical broad-spectrum intra-peritoneal antibiotics (Vancomycin and Gentamicin) were commenced. On day 5, he developed worsening abdominal pain. On examination, his blood pressure was 158/100 mmHg; heart rate 90/min, temperature 37.5 °C. His abdomen was tender on palpation. There was no evidence of PD catheter exit site infection or any tunnel infection. The CRP was 456.9 mg/L, and WCC was 12.3 × 10^6^/L. He was admitted to the renal unit and treatment was escalated to intravenous Gentamicin and Vancomycin, and subsequently to intravenous Meropenem on day 7 in view of persistent symptoms and raised inflammatory markers. He underwent rapid PD fluid exchanges to help relieve abdominal pain. On day 8, his CRP was 534 mg/L, WCC was 20.5 × 10^6^/L with a temperature of 39.8 °C. He underwent emergency surgical PD catheter removal with peritoneal washout. Intra-operative findings did not reveal any bowel perforation. A haemodialysis catheter was inserted, and regular haemodialysis (HD) was commenced. On day 8, acid-fast bacilli [AFB] were grown from the PD fluid sample, which was confirmed by the National Mycobacterium Reference Laboratory through whole-genome sequencing to be *Mycobacterium abscessus*. In view of this, he was commenced on quadruple therapy with intravenous Amikacin, Clarithromycin, Tigecycline and Imipenem with cilastatin. He developed new QTc prolongation on the electrocardiogram (ECG) on day 16, which was attributed to Clarithromycin, so this was therefore discontinued. Furthermore, he developed acute hepatic impairment which resolved following cessation of Tigecycline on day 26.

**Fig. 1:**
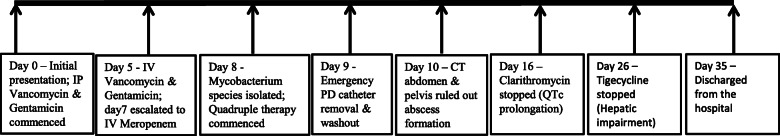
Timeline of inpatient stay

He remained clinically well on dual therapy with intravenous Amikacin and Imipenem and was discharged on day 35 (Fig. 1). A peripherally inserted central venous catheter was placed, to facilitate daily intravenous antibiotics as an outpatient. He continued on antibiotics for a total of 5 months. Linezolid was added during the last 6 weeks of therapy. He had a regular review by the Audiologists, but in spite of close monitoring to maintain the Amikacin levels within the therapeutic range, he has sustained a degree of sensorineural hearing loss.

## Discussion and conclusion

*M.abscessus* is a nontuberculous mycobacterium (NTM). It is one of the most clinically relevant, rapidly growing mycobacteria, which are environmental organisms that usually grow in culture within 1 week. *M. abscessus* has been frequently isolated from water, soil, domestic and wild animals [6]. Although not highly virulent, *M.abscessus* is known to cause disseminated infection in immunocompromised hosts, and bacteraemia can occur in the context of dialysis catheter use [7]. It is a well-documented cause of pulmonary infection in patients with structural lung disease such as cystic fibrosis, and can cause skin and soft tissue infections in hospitalised post-surgical patients [8, 9].

### Diagnosis

The diagnosis of NTM and *M.abscessus* PD peritonitis can be challenging. NTM can cause culture-negative infection, and it is interesting to note that in 4 out of 16 reported cases, the development of PD peritonitis was preceded by catheter replacement for the management of what was deemed to be culture-negative ESI or TI [3, 10, 11]. Clinicians should, therefore be vigilant with regards to possible NTM infection in patients with culture-negative PD peritonitis, particularly in the context of non-responsiveness to standard anti-microbial therapy [5, 12].

Our patient was diabetic and on PD for his kidney failure. The presence of end-stage renal failure (ESRF) is associated with impairment of the innate and adaptive immune system [13]. Diabetes mellitus was present in 27.5% of cases of NTM related PD peritonitis in one systematic review, suggesting that metabolic disease may pre-dispose to NTM infection [5]. *M.abscessus* has a predisposition to create biofilms, colonise and infect catheters [7]. Consequently, all previously documented cases of *M.abscessus* PD peritonitis have required catheter removal for effective source control (Table 1). The intrinsic resistance of *M.abscessus* to classical anti-tuberculous drugs as well as most other available broad-spectrum antibiotics limits the choice of options for therapy [20] and poses a significant challenge with regards to treatment.

**Table 1:** Summary of previously reported cases of *M.abscessus PD peritonitis*

Case number	Age: sex	Duration of PD	Time to culture	Positive AFB stain	Removal of PD catheter	Antibiotics and duration	Clinical outcome	Prior ESI, TI or abdominal intervention prior.	Country of reported case
1 [14]	67 M	12 months			Y	Amikacin, Clarithromycin (3 months)	Converted to HD		USA
2 [12]	70 M	36 months	9 days	N	Y	Amikacin, Clarithromycin Clarithromycin stopped (hepatic impairment)	Converted to HD. Died 6 weeks into treatment, acute coronary syndrome.	Y - Umbilical hernia repair	China
3 [12]	50 M	6 months	6 days	N	Y	Ciprofloxacin, Clarithromycin (3 months)	Converted to HD	Y – culture negative ESI	India
4 [12]	56 M	36 months	8 days	N	Y	Amikacin, Clarithromycin	Converted to HD. Re-admission with catheter related infection and sepsis 2 weeks after discharge. Died from acute coronary syndrome.	Y- *Pseudomonas* ESI	China
5 [12]	60 M	46 months	6 days	Y	Y	Amikacin (6 weeks) Clarithromycin (3 months) Amikacin stopped as developed sensorineural hearing loss.	Temporarily converted to HD. Re-established on PD after 3 months.	N	China
6 [15]	60F	60 months	6 days	Y	Y	Amikacin, Clarithromycin (8 weeks)	Converted to HD	Y – accidental removal of Tenckhoff catheter	Saudi
7 [15]	64F	12 months	4 days	Y	Y	Clarithromycin (12 weeks)	Converted to HD	N	Saudi
8 [16]	39F	60 months	Not disclosed	Not disclosed	Y	Meropenem (4 weeks), Amikacin (8 weeks), Cefoxitin (28 weeks)	Converted to HD. Intra-abdominal collection requiring drainage.	Recurrent P.*aeruginosa* ESI 2004–2007	Hong Kong
9 [16]	45 M	3 months	Not disclosed	Not disclosed	Y	Amikacin (4 weeks), Azithromycin 6 weeks) Moxifloxacin (20 weeks)	Temporarily converted to HD. Re-established on PD after 9 months.	N	Hong Kong
10 [17]	49 M	Not disclosed	Not disclosed	Not disclosed	Y	Cephazolin, Gentamicin	Converted to HD. Died after 5 months (sclerosing peritonitis)		Australia
11 [17]	40F	Not disclosed	Not disclosed	Not disclosed	Y	Amikacin, Cefoxitin, Vancomycin, Gentamicin, Flucloxacillin	Converted to HD		Australia
12 [17]	65 M	Not disclosed	Not disclosed	Not disclosed	Y	Clarithromycin, Vancomycin, Ticarcillin and clavulanic acid, Gentamicin	Converted to HD		Australia
13 [18]	44F	96 months	28 days	Y	Y	Clarithromycin (165 days), Amikacin (68 days), Meropenem (165 days), Levofloxacin (111 days)	Converted to HD. Died after 8 months (renal haemorrhage and retroperitoneal infection).	Y – Catheter replacement for refractory exit site infection. Commenced on anti-tuberculous drugs post AFB.	Taiwan
14 [18]	58F	60 months	10 days (treatment commenced day 3)	Y	Y	Clarithromycin (165 days), Amikacin (50 days), Imipenem (70 days), Ciprofloxacin (217 days), Doxycycline (180 days).	Converted to HD. Abscess formation in abdominal wall requiring debridement × 2.	Y – Catheter replacement 3 weeks prior for tunnelled line infection.	Taiwan
15 [11]	51F	14 months	Not disclosed	Y	Y	Clarithromycin (7 weeks) Ciprofloxacin (3 weeks) Amikacin (3 weeks)	Converted to HD. Disseminated cutaneous infection requiring surgical debridement	Y – PD catheter replacement 8 days prior (persistent culture neg. Tunnel infection)	Japan
16 [19]	56 M		33 days	N	Y	Imipenem (4 weeks) Clarithromycin (6 months)	Converted to HD. Infection post catheter insertion.	Y – Re-insertion of PD catheter (refractory ESI and TI presumed to be methicillin resistant coagulase negative staphylococcus)	Japan

A systematic review of 57 cases of NTM PD peritonitis found that the time from onset of symptoms to the initiation of appropriate treatment was 4 weeks [5]. This delay is multi-factorial. Firstly, the available data do not demonstrate any factor unique to *M.abscessus* PD peritonitis compared with more common organisms. The symptoms and signs are indistinguishable from conventional PD peritonitis or tuberculous peritonitis [4, 21]. All cases of *M.abscessus* PD peritonitis first received empirical antibiotics and in one case, subsequently received anti-tuberculous therapy following a positive acid-fast bacillus (AFB) stain [22]. Secondly, the utility of AFB staining in such cases is debatable, as the sensitivity of this test is not high enough [23]. In one particular study, AFB staining was positive in only four out of 10 cases of *M.abscessus* peritonitis [4]. Most centres do not perform AFB staining routinely as it does not distinguish between tuberculous mycobacteria and NTM, thus providing no further information regarding anti-microbial sensitivities [24, 25]. As a consequence, the diagnosis is reliant on bacterial culture alone, which leads to delays in the initiation of appropriate treatment and may likely result in worse outcome in such cases [26].

### Treatment

The management of *M.abscessus* PD peritonitis is complex. Catheter removal appears to be essential to recovery in the acute setting, with long term management reliant on anti-microbial therapy [12, 17]. *M.abscessus* is a multi-drug resistant organism, and as a consequence, the antibiotic choices are limited. One report investigated resistance and susceptibility criteria of *M.abscessus* in various published cases and showed that resistance rates for Quinolones, Doxycycline and Imipenem, were considerably high, while resistance rates for Cefoxitin (15.1%) and Amikacin (7.7%) were low. Clarithromycin showed variable resistance rates ranging from 0 to 38%. As a consequence, treatment should be based on the susceptibility profile of the bacterium isolated from individual patients [18].

It has been recommended that non-pulmonary *M.abscessus* infection should be treated for between 4 and 6 months [27]. There is no consensus, however, for the optimal duration of the treatment for *M.abscessus* PD peritonitis and this is reflected in the variability of treatment regimens observed in the reported cases, where treatment duration ranged from 7 weeks to 28 weeks with a variety of drug combinations used based on individual sensitivities.

The challenges posed by the administration of long-term antibiotics should also be recognised. In our case, quadruple therapy was not tolerated due to the development of hepatic impairment with Tigecycline and QTc prolongation with Clarithromycin. Similar complications were observed in previous cases with Amikacin (sensorineural hearing loss) and Clarithromycin (hepatic impairment) [12]. The optimal duration of anti-microbial therapy is unclear at present but is likely to be at least 4 months [27].

Overall, outcomes from *M.abscessus* PD peritonitis appear to be worse than for conventional organisms. A 10-year retrospective cohort study of PD peritonitis in an Australian population found that the risk of catheter removal varied between < 20 to > 40% depending on the causative organism [17]. The risk of changing over to HD was estimated to be between 5 to 20% [1]. In previously reported cases of *M.abscessus* PD peritonitis, 100% of patients were converted to HD [6]. In only two of these cases were the patients successfully converted back to PD, suggesting that the risk of PD failure is higher with *M.abscessus* infection compared with conventional organisms.

In summary, we present, to our knowledge, the first reported case of *M.abscessus* PD peritonitis in the United Kingdom. It was managed with catheter removal, a prolonged course of intravenous broad-spectrum antibiotics and conversion to HD. Clinicians should have a high index of suspicion of *M.abscessus* in cases of non-resolving PD related infections, or culture-negative peritonitis. Catheter removal appears to be essential for clinical recovery, and the switch to HD may be permanent.

## Data Availability

Not applicable.

## Abbreviations

AFB: Acid-fast bacillus
CRP: C-reactive protein
ECG: Electrocardiogram
ESI: Exit-site infection
ESKD: End-stage kidney disease
HD: Haemodialysis
*M abscessus*: *Mycobacterium abscessus*
*M.tuberculosis*: *Mycobacterium tuberculosis*
*M.leprae*: *Mycobacterium leprae*
NTM: Nontuberculous mycobacteria
PD: Peritoneal dialysis
TI: Tunnel infection
UK: United Kingdom
WCC: White cell count
